# Reliable Detection of Paternal SNPs within Deletion Breakpoints for Non-Invasive Prenatal Exclusion of Homozygous α^0^-Thalassemia in Maternal Plasma

**DOI:** 10.1371/journal.pone.0024779

**Published:** 2011-09-29

**Authors:** Ti-Zhen Yan, Qiu-Hua Mo, Ren Cai, Xue Chen, Cui-Mei Zhang, Yan-Hui Liu, Ya-Jun Chen, Wan-Jun Zhou, Fu Xiong, Xiang-Min Xu

**Affiliations:** 1 Department of Medical Genetics, School of Basic Medical Sciences, Southern Medical University, Guangzhou, Guangdong, People's Republic of China; 2 Center for Prenatal Diagnosis, Liuzhou Municipal Maternity and Child Healthcare Hospital, Liuzhou, Guangxi, People's Republic of China; 3 Center for Prenatal Diagnosis, The Affiliated Zhongshan Boai Hospital of Southern Medical University, Zhongshan, Guangdong, People's Republic of China; 4 Department of Medical Laboratory, The Affiliated Donghua Hospital of Sun Yat-Sen University, Dongguan, Guangdong, People's Republic of China; 5 Center for Prenatal Diagnosis, Shaoguan Municipal Maternity and Child Healthcare Hospital, Shaoguan, Guangdong, People's Republic of China; Stanford University, United States of America

## Abstract

Reliable detection of large deletions from cell-free fetal DNA (cffDNA) in maternal plasma is challenging, especially when both parents have the same deletion owing to a lack of specific markers for fetal genotyping. In order to evaluate the efficacy of a non-invasive prenatal diagnosis (NIPD) test to exclude α-thalassemia major that uses SNPs linked to the normal paternal α-globin allele, we established a novel protocol to reliably detect paternal SNPs within the (−−^SEA^) breakpoints and performed evaluation of the diagnostic potential of the protocol in a total of 67 pregnancies, in whom plasma samples were collected prior to invasive obstetrics procedures in southern China. A group of nine SNPs identified within the deletion breakpoints were scanned to select the informative SNPs in each of the 67 couples DNA by multiplex PCR based mini-sequencing technique. The paternally inherited SNP allele from cffDNA was detected by allele specific real-time PCR. A protocol for reliable detection of paternal SNPs within the (−−^SEA^) breakpoints was established and evaluation of the diagnostic potential of the protocol was performed in a total of 67 pregnancies. In 97% of the couples one or more different SNPs within the deletion breakpoint occurred between paternal and maternal alleles. Homozygosity for the (−−^SEA^) deletion was accurately excluded in 33 out of 67 (49.3%, 95% CI, 25.4–78.6%) pregnancies through the implementation of the protocol. Protocol was completely concordant with the traditional reference methods, except for two cases that exhibited uncertain results due to sample hemolysis. This method could be used as a routine NIPD test to exclude gross fetal deletions in α-thalassemia major, and could further be employed to test for other diseases due to gene deletion.

## Introduction

α-Thalassaemia is the most common monogenic inheritance disorder in tropical and subtropical areas in the world with a carrier frequency reaching as high as 10–15% in Southeast Asia, the Indian subcontinent, and Southern China [1],[2]. This inherited anemia syndrome mainly results from gross deletions of the human α-globin gene cluster mapped to chromosome 16p13.3, and thus is characterized by a reduction or complete suppression of α-globin chain synthesis [1],[3]. Deletion of the double α-globin gene is designated as α^0^-thalassaemia, such as −−^SEA^, −−^FIL^ and−−^THAI^, which is more prevalent in southeastern Asia including southern China. This deletion can result in a fatal condition, hemoglobin (Hb) Bart's hydrops fetalis, when the deletion is homozygous [4]. When both parents are α^0^-thalassaemia carriers, each pregnancy has a 25% risk for Hb Bart's hydrops. An affected fetus can be effectively detected by prenatal diagnosis for at-risk couples by fetal sampling via amniocentesis and chorionic villus sampling (CVS). However, such traditional techniques are invasive and carry a small but significant risk of miscarriages (0.5–1%) [5].

Fetal nucleated red blood cells [6]–[9], or cell-free fetal DNA (cffDNA) [10]–[13], that exist in maternal circulation during pregnancy offers a potential source of fetal material for non-invasive prenatal diagnosis (NIPD) of the fetal genetic status. Reliable NIPD tests using cffDNA has recently been used to detect paternally inherited genetic traits, such as Y-linked disorders and rhesus D [13]. A panel of highly sensitive technologies to detect paternally inherited fetal point mutations [14], has been developed and NIPD using cffDNA has been applied to a group of single gene disorders including β-thalassemia [15]–[18], cystic fibrosis [19], and congenital adrenal hyperplasia (CAH) [20], and has been used on the targeted genes in couples who carry different mutations in particular. It has been demonstrated that an alternative approach for detection of different SNPs linked to the mutant allele in parents with identical point mutations is potentially useful in non-invasive diagnosis using cffDNA [21].

It is technically challenging to reliably detect large fetal deletions from cffDNA in maternal plasma when couples share the same deletion due to the lack of specific markers for fetal genotyping. In addition, the fragmented nature of cffDNA is not conducive to efficient detection of large deletions using a PCR-based technique [22]. Recently, Ho et al. reported a method for identification of non-deleted paternally inherited fetal alleles in cell-free DNA (cfDNA) using two microsatellite markers within deletion breakpoints in NIPD for Hb Bart's syndrome [22], [23]. The number of polymorphic STRs in the region of interest is a disadvantage as it is limited, and therefore the method is only applicable for one-third of pregnancies [23]. Using α-thalassaemia as a diagnostic target, we present a novel strategy for the noninvasive prenatal diagnosis of a homozygous deletion using multiplex SNP discrimination of fetal DNA from maternal plasma. In this study we established a protocol for the reliable detection of a panel of paternal SNPs within the (−−^SEA^) breakpoints in cfDNA samples and evaluated 67 pregnancies at risk for α-thalassemia major; moreover, we demonstrated the feasibility and reliability of this NIPD method.

## Materials and Methods

### Sample collection

A total of 150 normal unrelated individuals and 150 αα/−−^SEA^ carriers were recruited for characterization of SNPs within the (−−^SEA^) breakpoints in the α-globin gene cluster (Accession NG_000006.1).To establish multiplex PCR-based mini-sequencing for simultaneous scanning of multiple SNPs, 40 standard sequenced samples were used in the study. 10 mL maternal blood samples were collected into two 5 mL EDTA blood collection tubes. Maternal plasma samples from 67 pregnant women at risk for α-thalassemia major were collected prior to invasive obstetrics procedures (8.5–25 weeks of gestation, mean 19.12 weeks, before chorionic villus sampling in the first trimester and before amniocentesis in the second trimester) between June 2008 and June 2010 in southern China. The patients' age ranged from 20 to 41 years with a mean of 27 years and median of 26 years. Both of the parents of each pregnancy carried an identical (−−^SEA^) deletion except for one case. The study was approved by the Ethics Committee of Nanfang Hospital, an affiliate of Southern Medical University, China. Detailed written informed consent was obtained from all participants.

### Blood processing and maternal plasma extraction

The buffy coat was collected from blood samples after centrifugation at 1600 g for 10 min, whereas maternal plasma was collected after an additional microcentrifugation at 16000 g for 10 min. Buffy coat DNA was extracted using a standard phenol-chloroform method. Four milliliters of maternal plasma from each pregnancy were used for DNA extraction using the QIAamp DNA blood Mini Kit (Qiagen, Crawley, UK) according to the manufacturer's instructions with minor modifications[19],[24]. In order to obtain efficient recovery of DNA without decreasing its concentration, some slight modifications of the elution protocols that were provided by the manufacturer were made following the previous procedure [19]. The yield of cfDNA from each maternal plasma sample could be increased when the maternal plasma volume processed the 200-µL aliquots limitation on manufacturers. In our procedure, 4 mL of each plasma from pregnant women was extracted by use of five Qiagen minicolumns by processing two 400-µL aliquots on each of them. The cfDNA immobilized on the five columns was manually eluted one by one with a total of 140 µL of autoclaved water and the isolated cfDNA from one maternal plasma sample was in 70 µL final volumes. The CVS samples and amniotic fluid samples were handled and analyzed using the multiplex PCR-based mini-sequencing and the α-thalassemia deletion Gap-PCR as previously described by Xiao *et al* [25] in four clinical centers.

### Diagnostic strategy

**Figure 1:**
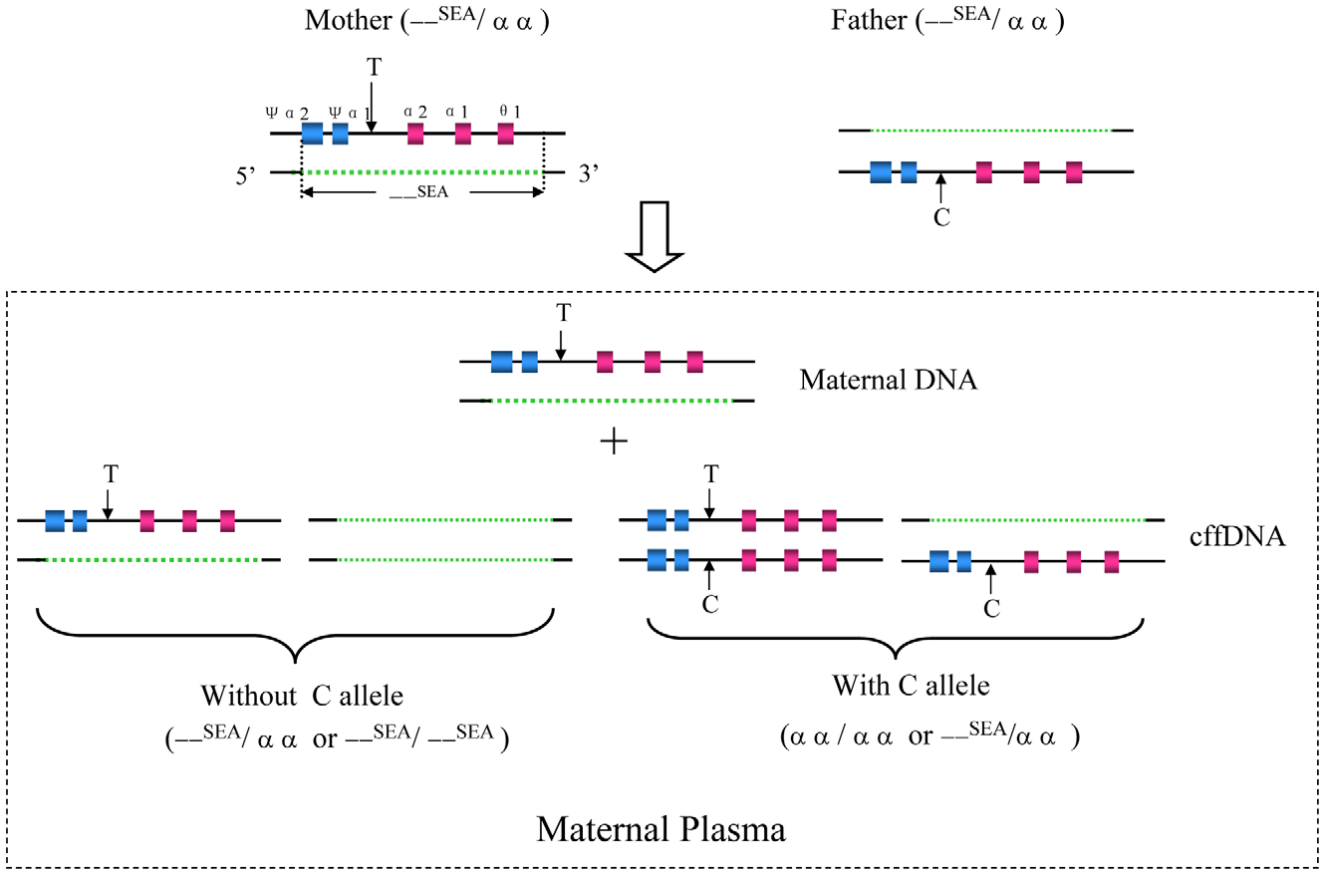
Strategy for the non-invasive prenatal exclusion of homozygous (−−^SEA^) deletion in maternal plasma. Schematic drawing showing the basic principle of our strategy for the non-invasive prenatal exclusion of homozygous (−−^SEA^) deletion based on SNP typing linked to the paternal-normal α-globin allele. The normal α-globin gene cluster and corresponding deleted region of (−−^SEA^) allele are denoted by the colored box with black line and the orange dotted line, respectively. The at-risk heterozygous couples sharing the same (−−^SEA^) deletion are shown on the top and the possible consequences of NIPD for the fetus are demonstrated by the bottom panel of this figure. The arrows indicate an informative SNP identified within the deletion breakpoints that can be used to differentiate between the mother's (with T allele, downward arrows) and the father's (with C allele, upward arrows) normal allele. If C allele is not detected in the cffDNA fraction of maternal plasma, the fetus may either be heterozygous, those who inherit a normal maternal T allele and a deleted paternal allele, or homozygous for the (−−^SEA^) allele, those who inherit deleted alleles from both parents. In contrast, the presence of C allele in cffDNA denotes that the fetus inherits the normal paternal allele, and therefore, we can exclude the possibility of Hb Bart's syndrome for the fetus.

**Figure 2:**
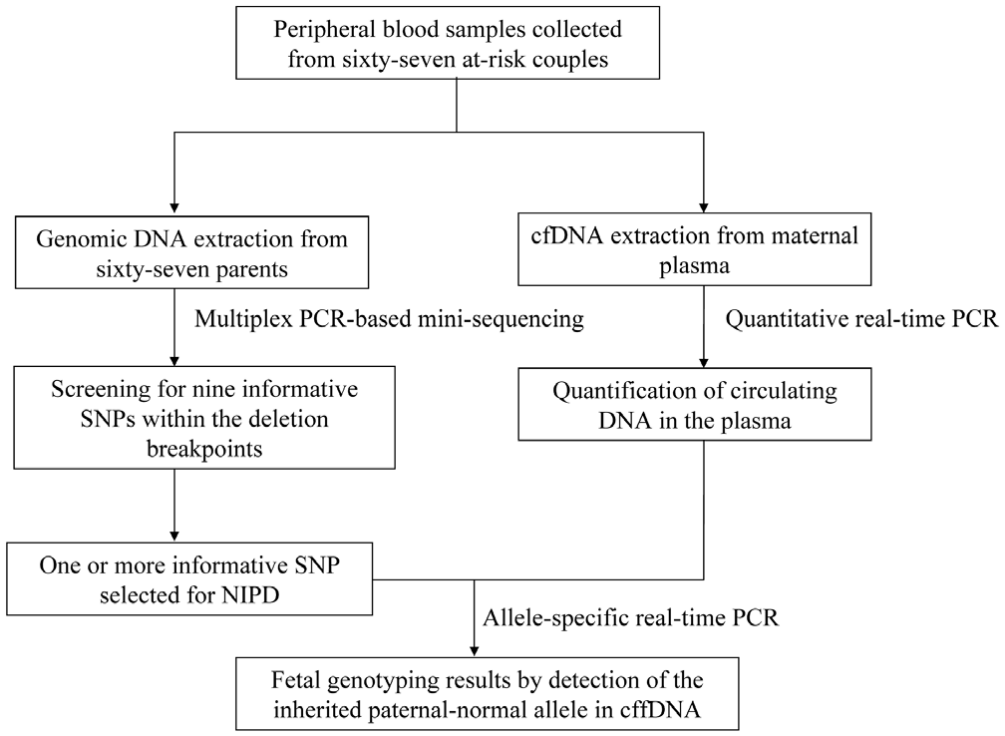
NIPD flow chart for exclusion of a large fetal deletion from cfDNA in maternal plasma.

In α-thalassaemia with a cis double gene deletion, the presence of the paternally inherited SNP allele in the maternal plasma would suggest that fetus had inherited the paternal normal α-globin allele and thus would not manifest hemoglobin Bart's hydrops fetalis; the diagnostic strategy is proposed as shown in Figure 1. The NIPD flow chart for exclusion of a large fetal deletion (the −−^SEA^ deletion) from cfDNA in maternal plasma is illustrated in Figure 2. A total of 67 at-risk heterozygous couples with the (−−^SEA^) deletion were recruited for this study. Initially, peripheral blood samples were collected from each of the at-risk couples, white blood cells from all couples were then used to isolate genomic DNA, and maternal plasma was used to isolate cfDNA; subsequently, quantitative real-time PCR (Q-PCR) was performed to quantify total cfDNA and cffDNA in the maternal plasma. Secondly, multiplex PCR-based mini-sequencing was used to identify informative markers using genomic DNA from both parents by screening for nine previously known SNPs within the deletion breakpoints. Finally, an allele-specific real-time PCR method was used to detect the normal paternally inherited fetal allele using cfDNA in maternal plasma, according to the detailed information on one or more specific SNPs (generally two sites were selected) that differ in the maternal and paternal genomes. The NIPD results from analyses on all families with informative SNPs were compared with results obtained from conventional invasive prenatal diagnosis (CIPD) in a blinded manner. The CIPD results were uncovered only once the genotypes obtained by NIPD were scored.

### Screening for informative SNPs within deletion breakpoints

Genomic DNA from the 67 couples was used to select informative markers useful for further non-invasive testing. Informative SNP markers were identified by multiplex PCR amplification using a Perkin Elmer Gene Amp PCR system 9600 thermal cycler (Applied Biosystems, Foster city, CA), followed by mini-sequencing based on a fluorescent-labelled dideoxy single-base extension of an unlabeled oligonucleotide primer or primers that hybridize upstream of the SNP site. The analysis was performed using an ABI3730 Genetic Analyzer and GeneMapper v3.7 software. The specific primer sequences and PCR cycling conditions for the informative SNP markers are listed in Table 1. For the multiplex PCR amplification, 50 ng of genomic DNA was used as the template. The final volume was 20 µL, containing 10 pmol of primer mixture, 2 µL 10×PCR buffer (Mg^2+^ free, Invitrogen), 0.8 µL 50 mM MgCl_2_ (Invitrogen), 0.5 µL 10 mM dNTPs (Takara), and 1 U Platimum Taq DNA polymerase (Invitrogen). After an initial incubation at 95°C for 15 min, the reaction was cycled for 30 s at 94°C, 30 s at 56°C, and 1 min at 68°C for 32 cycles, followed by a final extension for 30 min at 68°C. Excess primers and dNTPs were removed by the addition of 5 U shrimp alkaline phosphatase (SAP) and 2 U Exol to 15 µL of PCR product and incubation of the mixture at 37°C for 1 hour, followed by 75°C for 15 min. Following the PCR reaction, 5 µL was run as needed on a 1.5% agarose gel to check efficiency and specificity. Sequence of the single base extension primers including different polymeric T tails (Table 1). 2 µL of purified PCR product from parental samples and 2 µL from the controls were used as the template. The reaction was carried out in a final volume of 5 µL, containing 2.5 µL of SNaPshot ®Multiplex Ready Reaction Mix and 5 pmol of the specific unlabeled primers. The reaction was cycled for 10 s at 96°C, 5 s at 50°C, and 30 s at 60°C for 25 cycles. Excess nucleotides were removed by the addition of 1 µL (1 U/µL) SAP and 1 U CIP, and incubation of the mixture at 37°C for 1 h followed by 75°C for 15 min. Purified product from control DNA was diluted 1∶10 before being charged in the automated sequencer. For the analysis, 0.5 µL of purified product from the parent's samples and 1 µL from the controls were mixed with 9 µL of Hi-Di formamide and 0.5 µL of GeneScan 120 Liz size standard, followed by incubation of the mixture at 95°C for 5 min and then immediately frozen 5 min.

**Table 1:** The oligonucleotide sequences of Multiplex PCR amplification and extension primer sequences for the mini-sequencing reaction.

Marker ID	SNP ID	Marker position on NG_000006.1^a^	Multiplex PCR Sequence (5′to 3′)	Amplicon (bp)	ASPE Primer Sequence (5′to 3′)	Product length (bp)
1	rs2858935	g.26719G>C	F-primer: CCTCCTGGCAGAGCAGTAACTT	255	(T)_17_-TCCACACAGACGGGAACCCG	38
			R-primer: CCGCCCTGGCCTCCTTAT			
2	rs75368786	g.27606C>A	F-primer: CCGTGGTGCTGACCGAAAAA	315	(T)_28_-TGGCCTTGGTCTGTGCCTGT	49
			R-primer: TGGTTGGAAGCCCGATTCA			
3	rs2541675	g.29599A>G	F-primer: AGGCACCTCCTGGCAACTT	362	(T)_33_-CGGTCTGGGAGAAAGTTGGC	54
			R-primer: GGCCAAGTGAGAGGAAGGTC			
4	rs2974771	g.31921T>C	F-primer: CTACAACTACTGCCACAGGCTCT	136	(T)_31_-AAAATACCATCATACTGTAGATACC	57
			R-primer: GAATGGCGCAGAGCTGAAT			
5	rs2541669	g.33004C>T	F-primer: GTCACAGTGAACCACGACCTCT	235	(T)_38_-ATGACTTGGGGCTTAGCCAG	59
			R-primer: CTTTCCCTCTGGCGATAGTCA			
6	rs2238369	g.35483T>C	F-primer: GGAATCCATGCTGGGAAGTT	217	(T)_23_-GGTGGAGAGGACCCTGTCAC	44
			R-primer: GTGGAGAGGGGAGGGAACT			
7	rs11639532	g.36023G>A	F-primer: ACGGGGGAAGCATTGCTAA	539	(T)_26_-GGGGAAGCATTGCTAAGCT	46
8	rs2858942	g.36517A>C	R-primer: GGTGTGGACGAGGCATTCA′		(T)_32_-GCCCTCAGACTAACCCTGGTC	54
9	rs3760046	g.38757T>C	F-primer: CGCTGGACCCTAGAGTGCTT	314	(T)_38_-AGAGGTCCTCCCACATATGGG	60
			R-primer: CCGCACCCCTGATTTCATCT			

a GenBank accession number.

Abbreviations: SNP, single nucleotide polymorphism; bp, base pair.

### Quantification of circulating DNA in the plasma

Real-time Q-PCR was performed using primers and probe specific to the *GAPDH* gene in all pregnancies to determine the total amount of circulatory maternal plasma DNA. The male-specific *SRY* gene was used to monitor the presence of cffDNA and quantify cffDNA. This was achieved according to a method previously described by Birch *et al* [26]. The amount of circulatory maternal plasma DNA obtained from 67 pregnant women and cffDNA from mothers bearing male fetuses was calculated using real-time PCR threshold cycle values.

### Specificity and sensitivity of allele-specific real-time PCR

To define the sensitivity and specificity of allele-specific real-time PCR for detection of fetal DNA in the maternal plasma for this study, we conducted a series of experiments performed on artificial mixtures of mutant allele diluted into wild-type allele samples. For each SNP, 100 ng genomic DNA was successively diluted at 1∶1, 1∶10, 1∶100, 1∶1000, and 1∶10000 in water and used as a starting template for amplification. In addition, DNA samples that affect the ratio of paternal versus maternal alleles were prepared in PCR reaction tubes by mixing −−^SEA^/αα genomic DNA with maternal allele spiked in varying dilutions of 1∶1,1∶2, 1∶5, 1∶10, 1∶20, 1∶50, 1∶100, 1∶500, 1∶1000, 1∶5000 and 1∶10000 in −−^SEA^/αα genomic DNA with paternal allele diluents. The experiment was repeated twice. We optimized the specificity of each of the allele-specific assays for informative SNPs by assessing several factors including buffers composition, temperature, and length of PCR amplification cycles, together with the use of various oligonucletide primers [16], [19]. We then used the ΔC_T, (Paternal-Maternal)_ approach to measure the quantitative differences between the paternal allele and maternal allele [16]. We also used melting curve analysis to monitor the presence of the paternal allele.

### Detection of paternally inherited fetal SNP allele in maternal plasma

Allele specific real-time PCR was performed to detect the paternally inherited normal allele in the presence of an excess of the corresponding maternal sequence. As all informative SNP artificial experiments are functional, the analysis to detect paternally inherited fetal allele by allele specific real-time PCR in the maternal plasma was performed on a Thermocycler sequence detector (Stratagene Mx3005; La Jolla, CA, USA). For AS-PCR analysis, 10 µL of DNA isolated from maternal plasma was used in the PCR. Maternal plasma DNA was amplified in a final reaction volume of 25 µL containing 10 µM wild type primer or mutant primer 0.25 µL, 10 µM common primer 0.25 µL (Table S1), and 12.5 µL SYBR® Green Real-time PCR Master Mix (Applied Biosystems, Foster city, CA). The assay protocol started with an amplification procedure consisting of a denaturation step of 30 sec at 95°C, 55 amplification cycles of 10 s at 95°C, 10 s at annealing temperature for each SNP marker (Table S1), and 20 s at 72°C; and this procedure was followed by a melting analysis procedure consisting of a denaturation step of 1 min at 95°C, and a stepwise temperature increase from 60°C to 95°C at 0.5°C/step with 5 s detection for each step. The fluorescence from SYBR channel was recorded at each step during the annealing and the melting analysis procedure.

### Statistical analysis

Statistical analyses were conducted using SPSS 14.0 (Lead Technologies. Inc., Chicago, IL, USA). *P*<0.05 values were considered statistically significant.

## Results

Allele frequencies of 16 SNP markers obtained from 450 chromosomes in Southern Chinese are listed in Table 2. Nine SNPs with minor allele frequency (MAF) >0.15 were considered to be informative and were used in our protocol. Three SNP markers (rs2514675, rs2974771 and rs2858942) were highly informative (MAF>0.45). The locations of the nine SNPs are in the common region between the 5′ and 3′ end breakpoints of three α-thalassaemia double-gene deletions as shown in Figure S1 and Table S2.

**Table 2:** Allele frequencies of 16 SNP markers in 150 normal unrelated individuals and 150 heterozygote samples.

Marker ID	SNP ID	Marker position on NG_000006.1^a^	Number of markers with allele				Allele frequency of	
			Major		Minor		Major	Minor b
			Normal	heterozygote	Normal	heterozygote		
1	rs2858935	g.26719G>C	219	109	81	41	0.73	0.27
2	rs75368786	g.27606C>A	250	128	50	22	0.84	0.16
3	rs2541675	g.29599A>G	155	79	145	71	0.52	0.48
4	rs2974771	g.31921T>C	145	86	155	64	0.51	0.49
5	rs57397665	g.31990C>G c	145	86	155	64	0.51	0.49
6	novel	g.32905C>T	293	149	7	1	0.98	0.02
7	rs2541669	g.33004C>T	224	96	76	54	0.71	0.29
8	rs2362746	g.34247G>T	280	146	20	4	0.95	0.05
9	novel	g.34951G>A	295	149	5	1	0.99	0.01
10	rs2238369	g.35483T>C	215	98	85	52	0.70	0.30
11	rs223830	g.35511G>A	278	146	22	4	0.94	0.06
12	rs11639532	g.36023G>A	242	118	58	32	0.80	0.20
13	novel	g.36340G>A	296	150	4	0	0.99	0.01
14	rs2858942	g.36517A>C	164	84	136	66	0.55	0.45
15	rs3760046	g.38757T>C	252	130	46	20	0.85	0.15
16	rs1203834	g.42405C>T	260	145	40	5	0.90	0.10

a GenBank accession number.

b Those markers that minor allele frequency exceeded 0.15 were selected for informative markers.

c g.31990C>G was completely linked to the g.31921T>C, therefore we selected one of them for further analysis.

**Figure 3:**
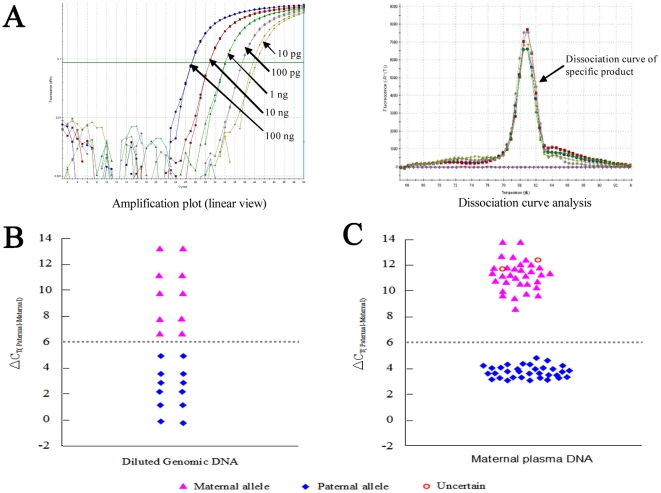
The sensitivity and specificity of allele-specific real-time PCR method used for NIPD. Panel A shows one set of representative data from analysis of g.31921 T>C marker by allele-specific real-time PCR, in which the SNP profiling of artificial model samples is exported by amplification blot (left) and dissociation curve analysis (right). Our results showed superior sensitivity and specificity in all dilutions (see materials and methods), indicated by the arrows for each test, with a sensitivity of detection of 2 copies of the target sequence. Panel B shows the quantitative difference between the respective C_T_ values (ΔC_T_) of maternal alleles (C_T, maternal_) and paternal alleles (C_T, paternal_) in analysis of g.31921T>C artificial model samples (see Materials and Methods). These results indicated a clear discrimination of the paternal allele from the maternal allele on experimental serial dilutions. Following the ΔC_T,(paternal-maternal)_ analysis, the maternal allele cluster is above a ΔCT value of 6, while the paternal allele cluster is at a value of less than 6, thus the arbitrarily assigned ΔCT cut-off value (gray dotted line) was used to distinguish between the presence of maternal from paternal alleles in the present study. Panel C shows the NIPD results obtained from detection of 65 at-risk fetus using 9 SNP markers in cfDNA as paternal-normal marker by allele-specific real-time PCR. Among 65 samples tested, correct appraisal of the presence (n = 33) or absence (n = 30) of the paternal-normal allele. Two cases labeled uncertain due to sample hemolysis by mishandling in the process of blood transport.

The mean concentration of cfDNA obtained from maternal plasma of 65 pregnancies was 611.63 genome-equivalents per milliliter (GE/mL); the mean concentration of fetal DNA in 31 plasma samples from mothers bearing male fetuses was 47.35 GE/mL. The concentration of circulating DNA obtained from maternal plasma of 65 pregnancies was within a certain range 87.8–1556 GE/mL (Table S3). On the basis of our experience with AS-PCR, it is possible to detect certain types of genetic alterations present at amount of 2 copies in a PCR tube (Panel A in Figure 3). In practice, we used 50 genome-equivalents of cfDNA as a lower cut-off value. The amount of input DNA (obtained from 4-mL maternal plasma samples) much exceeded 50 genome-equivalents to permit accurate analysis. They would be sufficient for the real-time AS-PCR assay to function reliably. In addition, the majority of whole blood samples (97%) were processed directly in four clinical centers and the plasma was sent frozen to our center by commercial express courier service. Two samples (case 21 and 38) were sent as whole blood by commercial express courier service. They were placed frozen gel packs directly on tubes of blood, therefore resulted in hemolysis. Based on previously reported results by Birch *et al* [26], these two cases had to be excluded because the amount of cfDNA exceed 49940 genome equivalents/mL and was substantially higher than would be expected in maternal samples during pregnancy (Table S3).

This method involved multiplex PCR-based mini-sequencing to scan for informative SNPs followed by allele-specific real-time PCR for NIPD results. The mini-sequencing assay demonstrated feasibility and reliability in simultaneously genotyping nine SNPs, and exhibited suitability for high-throughput SNP scanning of genomic DNA samples by multiple PCR-based fragment analysis. We validated the assay using a group of previously sequenced DNA samples (20 unrelated normal individuals and 20 −−^SEA^/αα carriers). The SNP typing of each case was accurately defined by the mini-sequencing assay at each nucleotide position analyzed, exhibiting 100% concordance with the reference method. SNPs were then assessed in 67 couples, and one or more different SNP(s) within the deletion breakpoints between paternal and maternal alleles was detected in 97% of the couples (65 cases). The SNPs were informative and could be further used as candidate polymorphic sites linked to the paternal-normal allele. Two cases (family 15 and family 35) were non-discriminative as none of the SNPs were informative (Table 3 and Table S5). Because the α-globin genes are duplicate (α2 and α1) with a high degree of homology (as illustrated in Figure S1), SNP ID rs2541669 (g.33004C>T) is located within the Z2 box of α2-globin gene, which is homologous to the Z1 box of α1-globin gene; while SNP ID rs2238369 (g.35483T>C) is located within the X1 box of α1-globin gene, which is homologous to the X2 box of α2-globin gene. The multiplex PCR of these two markers respectively yield two amplicons with identical length. The mini-sequencing amplicons of these two markers show the presence of two peaks of amplification in heterozygous (−−^SEA^) samples whereas all SNPs tested should be hemizygous. However, such expected detection results do not influence our ability to correctly type SNPs for each of these two sites. As illustrated in panel A of Figure S2-family 7A, g.33004 T from father was detected by both T peak (red) and additional C peak (black), thus indicating a truly positive T allele in this case. The g.35483 T is similar to g.33004 T, with two peaks showing in mother.

**Table 3:** Summary of the results of the informative SNP alleles by the analysis of cell free fetal DNA in the maternal plasma.

Number of informative polymorphisms	No. of cases	Correct detection of cases/Total No. of cases		Sensitivity (%)	Specificity (%)
		Presence of paternal allele	Absence of paternal allele		
0	2	UI^a^	UI^a^	-	-
1	5	2/2	3/3	100	100
2	6	2/2	4/4	100	100
3	7	3/3	4/4	100	100
4	5	1/1	4/4	100	100
5	25	14/14	9/11^b^	100	81.8
6	15	11/11	4/4	100	100
7	2	0	2/2	100	100
Total (excluding uninformative cases)	65	33/33	30/32	100	93.8

a Two families are excluded due to lack of informative SNP markers. UI: uninformative.

b Two cases are classified as uncertain, due to mishandling the samples.

Reference DNA samples and reliable genotyping samples of recombinant mutant clones were obtained in order to develop the allele specific real-time PCR assay technology. Because a mixture of maternal and paternal alleles was present, a model to test the discrimination capability between paternal and maternal alleles was established by subtracting the respective CT values (ΔC_T_) of maternal alleles (C_T, Maternal_) and paternal alleles (C_T, Paternal_) using a serial of artificial model samples for each of the 9 SNPs. These assessments permitted establishment of tentative cut-off ΔC_T_ value that could serve to distinguish between samples in which the paternal inherited allele was absent and those samples in which it was present. Following the ΔC_T, (Paternal-Maternal)_ analysis, the presence of paternally inherited alleles was at a ΔC_T_ value of less than 6 (Table S4). This method permitted a clear discrimination between maternal and paternal allele, even with experimental conditions whereby the maternal allele was diluted in paternal allele of the order expected in maternal plasma samples (Figure 3). The results of the model allele specific real-time PCR experiment demonstrated our optimized protocol could detect one copy of paternal DNA in every 50 copies of maternal DNA (Table S4). As illustrated in figure 3A, the data from the analysis of the g.31921T>C marker by the allele-specific real-time PCR assay showed unequivocal results. This assessment permitted the establishment of a tentative cut off value that could serve to discriminate between the presence and absence of the paternal-normal allele in the clinical cfDNA samples (Figure 3B). Furthermore, based on this cut off value we were able to correctly determine the paternal-normal allele of cffDNA from all 65 cases, revealing the sensitivity and robust accuracy of this technique (Figure 3C).

As shown in Table 3, a total of 65 cases were examined for the paternal-normal allele and the presence of those normal allele was detected in 2 out of 5 cases for one discriminative SNP, 2 out of 6 cases for two discriminative SNPs, 3 out of 7 cases for three discriminative SNPs, 1 out of 5 cases for four discriminative SNPs, 14 out of 25 cases for five discriminative SNPs, 11 out of 15 cases for six discriminative SNPs, and 0 out of 2 cases for seven discriminative SNPs. Two uncertain results were obtained in 2 cases in this study due to sample hemolysis. Homozygosity for the (−−^SEA^) deletion was accurately ruled out in 33 out of 67 (49.3%, 95% CI 25.4–78.6%) pregnancies tested. The overall sensitivity for the nine paternal SNP markers detected reached 100% and the specificity was 93.8% (Table 3). NIPD of fetuses who were at-risk for α-thalassemia major in two families using our protocol are illustrated in Figure S2. Results for the paternal-normal allele in the maternal plasma from 63 families obtained from analyses in which our protocol was implemented were in concordance with results obtained by conventional prenatal diagnosis (Table S5).

## Discussion

In the present blind study of 67 high genetic risk couples we have shown that PCR-based DNA detection of paternal SNPs within the deletion breakpoints is a rapid and reliable technique for non-invasive prenatal diagnosis of deletional α-thalassemia. A group of informative SNP markers within the (−−^SEA^) breakpoints that differ in the maternal and paternal chromosomes dependent on the natural variation in the Chinese population studied have been identified. Furthermore, the SNP markers designed in the present strategy are available for exclusion of the paternal deleted chromosome of other two common deletion of −−^THAI^ and −−^FIL^ resulting in α-thalassemia in Southeast Asia [23] (Figure S1), thereby potentially extended to include testing for these deletions for NIPD applications. We demonstrated the feasibility of our newly developed method as 97% of the couples (65 cases) had one or more different SNP(s) within the breakpoints of deletions between paternal and maternal alleles. 2 of the 67 cases were non-informative, due to the inheritance of identical SNP markers in both parents. Therefore, it is possible to exclude nearly 50% (33/67) of the at-risk subjects tested by exclusion of the homozygous (−−^SEA^) deletion based on detection of the paternally inherited fetal normal allele. With a potential application in one-half of pregnancies our SNP-based assay thus generally overcomes the limitations of microsatellite markers, although our assay cannot be used to differentiate homozygous deletions from carriers in the remaining 50% of at-risk pregnancies. In some cases, it is possible to detect fetal genetic loci for disease-causing point mutations, as well as SNPs linked to paternally inherited fetal mutant alleles involved in disorders such as β-thalassemia [15]–[18],[21],[27], achondroplasia [28], and myotonic dystrophy [29], although this is highly technically challenging. However, there are very few NIPD applications that can analyze single-gene disorders caused by large deletions through a reliable approach using cffDNA in maternal plasma. This novel approach has been successfully applied to the detection of α-thalassemia in the present study, and could be expanded to other autosomal genetic diseases due to gene deletions, such as 22q11.2 deletion syndrome and deletional β-thalassemia.

In addition to the conventional extraction of cffDNA, the key element of the molecular test involved in our approach is the two-step protocol involving multiplex PCR-based mini-sequencing and allele specific real-time PCR. By the use of the former method, we have shown that it is possible to simultaneously detect nine SNPs within breakpoints of the deletion tested using genomic DNA from high-risk parental individuals. The results obtained from our random samples indicated that two to seven informative SNPs could be detected 89.55% of at-risk couples. Thereby, the test is easy to process since only one SNP is needed in the practical protocol of NIPD for detection of the paternally inherited fetal allele in maternal plasma. The latter is used to detect one informative SNP selected from the above several sites for direct exportation of cfDNA sample NIPD results. Another informative SNP sites can be used to provide double-check information on NIPD for the same plasma sample previously tested if it is necessary. Uncertain results were obtained in 2 cases in this study due to sample hemolysis that could lead to significantly increasing maternal DNA in the tested samples; thereby the amount of maternal DNA in the PCR sample can interfere with the assay. There were several factors observed to affect the sensitivity and reproducibility of the assay system, particularly PCR polymerase, hence AmpliTaq Gold LD polymerase (Applied biosystem, No. 4367659) was selected to guarantee consistent results and we were able to consistently detect 2 copies of paternal alleles without interference from the maternal sequences in the model PCR experiment (Figure 3A).

In our study, plasma samples were collected during 8.5–25 weeks of gestation (mean 19.12 weeks), representing a relatively high level of cffDNA, in which the paternally inherited fetal SNPs, masked by the background of maternal DNA, can be definitely detected through allele-specific real-time PCR, indicating that the sensitivity of the molecular test is able to meet the demands of NIPD for clinical samples. Due to the limited quantities of cffDNA isolated between 8 and 12 weeks of gestation in early pregnancy we believe that it will be important to further enhance detection of SNPs in the application of this technology. Our method could be improved for non-invasive exclusion of fetal large deletions in monogenic disorders by inclusion of an additional step of selective enrichment of cell-free fetal DNA in maternal plasma by size fractionation [30]. Moreover, we would enhance the accuracy of our non-invasive prenatal exclusion of homozygous α-thalassaemia by using SNPs that lie outside the (−−^SEA^) deletion breakpoints as controls in early pregnancy.

In conclusion, this is the first large scale study reporting a contribution of detecting paternally inherited normal alleles within deletion breakpoints from α^0^-thalassemia patient maternal plasma samples. Based on our results, which made use of a combination of multiplex-PCR based mini-sequencing and allele-specific real-time PCR, our strategy could be directly applicable for noninvasive prenatal prediction of homozygous α-thalassaemia by maternal plasma DNA analysis. The advantage of detecting these paternal SNPs within deletion breakpoints in deletional Mendelian disorders is that their presence could be used to exclude pregnancies at risk for these disorders, thereby obviating the need for an invasive prenatal diagnostic procedure. Furthermore, a decrease of these risk-associated procedures could be achieved for diagnosis of these common regional genetic disorders.

## Supporting Information

Figure S1 **Nine informative SNP markers are located within the breakpoints of −−^THAI^, −−^FIL^ and −−^SEA^.** The detailed locations of nine SNP markers are listed in Table S1 that accompanies the online version of this article. The α-globin genes are embedded within two highly homologous 4 kb duplication units. These regions are divided into homologous subsegments (X, Y, and Z) by non-homologous elements.
(TIF)

Figure S2 **Representative NIPD for α-thalassemia in two families performed using our protocol.** The relevant results of the multiplex PCR-based mini-sequencing chromatograms are shown in the two windows (mother in top, father in middle and fetus in bottom), which were obtained from screening for nine informative SNPs within the deletion breakpoints by single-step test, with the arrows indicating those SNPs that differ in the maternal and paternal genomes. Each of nine SNP markers were detected in the given position of amplicons in different size (the scale in base pair on horizontal axis), from left to right, they are g.26719C>G, g.27606C>A, g.29599A>G, g.31921T>C, g.33004C>T, g.35483T>C, g.36023G>A, g.36517A>C and g.38757T>C. Each fluorescent dye corresponds to a different nucleotide where blue represents G, green represents A, black represents C, and red represents T. The CVS and amniotic fluid samples were analyzed using the multiplex PCR-based mini-sequencing by four clinical centers. The NIPD results for two at-risk fetus using two informative SNPs by allele-specific real-time PCR are shown in panel B (the specific SNPs tested are indicated on the top of figure). The allele-specific SNP profiling of DNA samples are exported by amplification blot (left) or dissociation curve analysis (right), in which the corresponding arrows indicate each profiling of samples amplified from four different sources, with the corresponding specific bases marked in bracket, FDP = fetal DNA in plasma; MDP = maternal DNA in plasma; PDB = paternal DNA in blood; MDB = maternal DNA in blood; NAP = nonspecific amplification products from primer-dimers; NTC = no template control. As shown in family 7 of the figure, for g.29599A>G marker, C_T, Maternal_ = 31.52, C_T, Paternal_ = 35.41, ΔC_T,(paternal-maternal)_ = 3.84; for g.36517A>C marker, C_T, Maternal_ = 30.96, C_T, Paternal_ = 34.86, ΔC_T,(paternal-maternal)_ = 3.90. The cffDNA was identified as having inherited paternal-normal alleles (g.29599G allele and g.36517C allele) in this family, and therefore, the NIPD results above indicated an exclusion of homozygosity for α^0^-thalassemia in this fetus. However, in family 47 of the figure, for g.26719C>G marker, C_T, Maternal_ = 34.53, C_T, Paternal_ = 43.97, ΔC_T, (paternal-maternal)_ = 9.44; for g.31921T>C marker, C_T, Maternal_ = 34.94, C_T, Paternal_ = 44.41, ΔC_T, (paternal-maternal)_ = 9.47. The inherited paternal-normal alleles, g.26719G and g.31921C, was not found in the cffDNA in plasma from Family 47, thus indicating the possibility of heterozygous or homozygous for the (−−^SEA^) deletion for this fetus. The comparative CIPD results for two families using gap-PCR are shown in panel C. The gel electrophoresis of PCR amplified fragments from normal allele (1052 bp) and the (−−^SEA^) allele (740 bp). MA = 100 bp DNA Marker ladder (Takara), C = no DNA control, F = fetus (DNA from CVS or amniocentesis), P = paternal, M = maternal, S = standard control with wild-type, heterozygote and homozygote of the (−−^SEA^) deletion, from right to left. Genotypes of these two fetuses were confirmatively diagnosed as having αα/αα in Family 7 (1052 bp) and −−^SEA^/−−^SEA^ in Family 47 (740 bp).
(PDF)

Table S1 **Oligonucleotide sequences for Allele-Specific Real-time PCR.**
(DOC)

Table S2 **The location of nine SNP markers within (−−^SEA^) deletion breakpoint regions.**
(DOC)

Table S3 **The quantification results of maternal plasma DNA by Real-time Quantitative-PCR.**
(DOC)

Table S4 **Allele specific real-time PCR analysis of nine SNP markers using a serial of artificial model samples.**
(DOC)

Table S5 **Comparison between the analysis of circulating fetal DNA and invasive procedure (CVS or amniocentesis).**
(DOC)
