# Supplementary figures and images for: Reliable Detection of Paternal SNPs within Deletion Breakpoints for Non-Invasive Prenatal Exclusion of Homozygous α0-Thalassemia in Maternal Plasma
Source: PLoS One. 2011 Sep 29;6(9):e24779. doi: 10.1371/journal.pone.0024779 (PMC3182989; doi:10.1371/journal.pone.0024779)

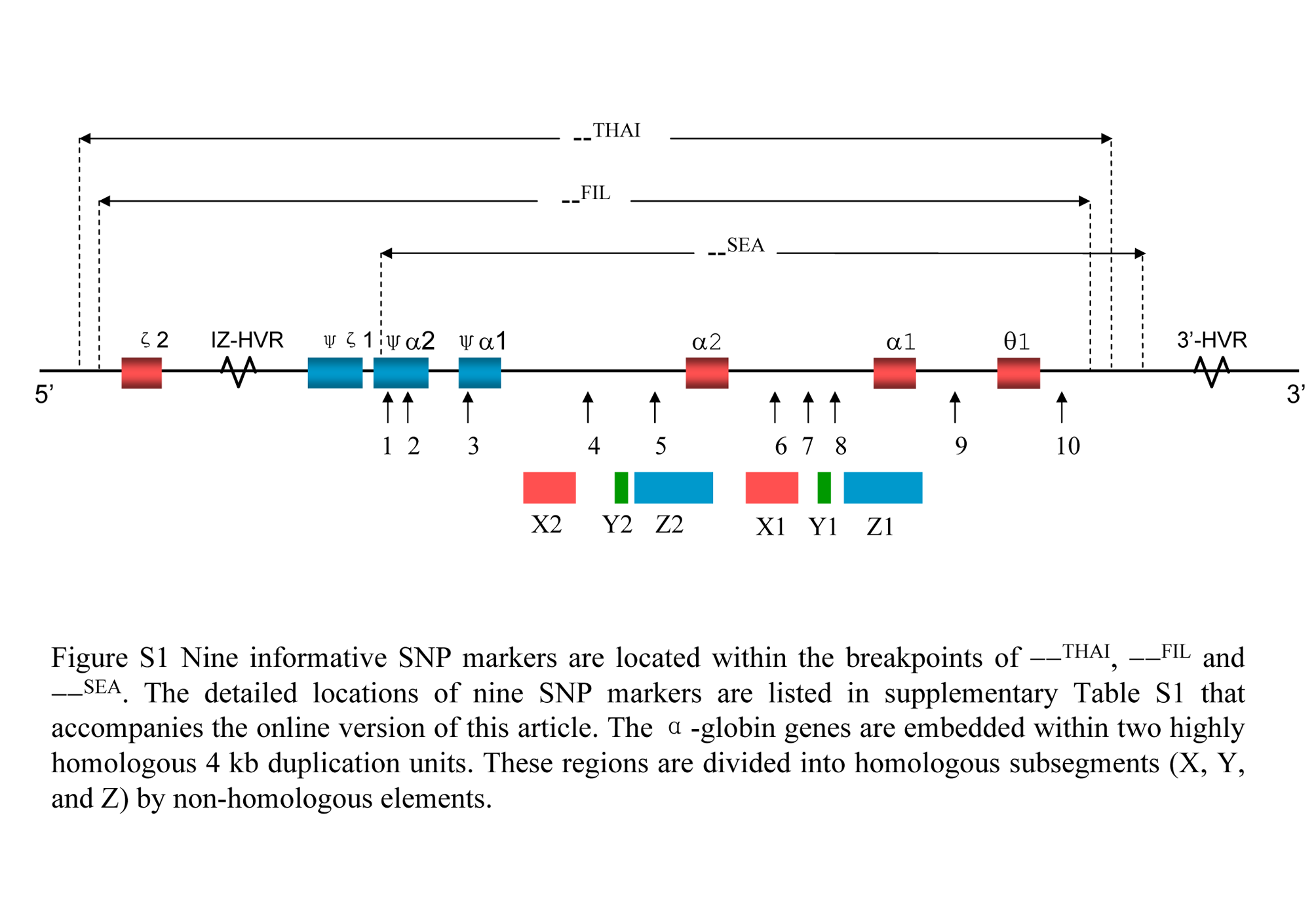

Supplement: Figure S1 — Nine informative SNP markers are located within the breakpoints of −−THAI, −−FIL and −−SEA. The detailed locations of nine SNP markers are listed in Table S1 that accompanies the online version of this article. The α-globin genes are embedded within two highly homologous 4 kb duplication units. These regions are divided into homologous subsegments (X, Y, and Z) by non-homologous elements. (TIF) [file pone.0024779.s001.tif]
