# Supplementary material for: Reliable Detection of Paternal SNPs within Deletion Breakpoints for Non-Invasive Prenatal Exclusion of Homozygous α0-Thalassemia in Maternal Plasma
Source: PLoS One. 2011 Sep 29;6(9):e24779. doi: 10.1371/journal.pone.0024779 (PMC3182989; doi:10.1371/journal.pone.0024779)
Supplement: Figure S2 — Representative NIPD for α-thalassemia in two families performed using our protocol. The relevant results of the multiplex PCR-based mini-sequencing chromatograms are shown in the two windows (mother in top, father in middle and fetus in bottom), which were obtained from screening for nine informative SNPs within the deletion breakpoints by single-step test, with the arrows indicating those SNPs that differ in the maternal and paternal genomes. Each of nine SNP markers were detected in the given position of amplicons in different size (the scale in base pair on horizontal axis), from left to right, they are g.26719C>G, g.27606C>A, g.29599A>G, g.31921T>C, g.33004C>T, g.35483T>C, g.36023G>A, g.36517A>C and g.38757T>C. Each fluorescent dye corresponds to a different nucleotide where blue represents G, green represents A, black represents C, and red represents T. The CVS and amniotic fluid samples were analyzed using the multiplex PCR-based mini-sequencing by four clinical centers. The NIPD results for two at-risk fetus using two informative SNPs by allele-specific real-time PCR are shown in panel B (the specific SNPs tested are indicated on the top of figure). The allele-specific SNP profiling of DNA samples are exported by amplification blot (left) or dissociation curve analysis (right), in which the corresponding arrows indicate each profiling of samples amplified from four different sources, with the corresponding specific bases marked in bracket, FDP = fetal DNA in plasma; MDP = maternal DNA in plasma; PDB = paternal DNA in blood; MDB = maternal DNA in blood; NAP = nonspecific amplification products from primer-dimers; NTC = no template control. As shown in family 7 of the figure, for g.29599A>G marker, CT, Maternal = 31.52, CT, Paternal = 35.41, ΔCT,(paternal-maternal) = 3.84; for g.36517A>C marker, CT, Maternal = 30.96, CT, Paternal = 34.86, ΔCT,(paternal-maternal) = 3.90. The cffDNA was identified as having inherited paternal-normal alleles (g.29599G allele and g [file pone.0024779.s002.pdf]

A

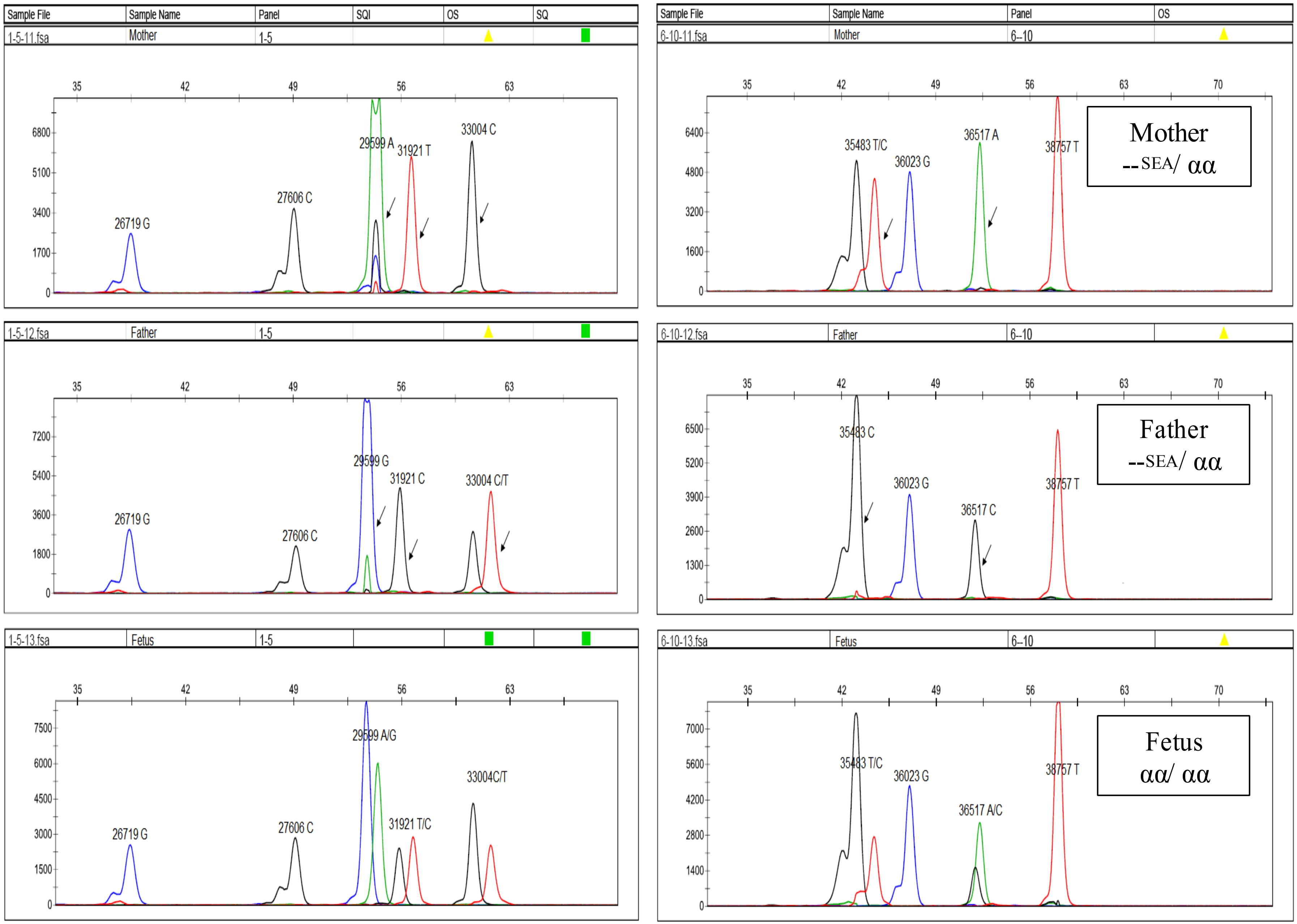
Sample File
Sample Name
Panel
SQI
OS
SQ
1-5-11.fsa
Mother
1-5
26719 G
27606 C
29599 A
31921 T
33004 C
1-5-12.fsa
Father
1-5
26719 G
27606 C
29599 G
31921 C
33004 C/T
1-5-13.fsa
Fetus
1-5
26719 G
27606 C
29599 A/G
31921 T/C
33004C/T
6-10-11.fsa
Mother
6–10
35483 T/C
36023 G
36517 A
38757 T
Mother
--SEA/ αα
6-10-12.fsa
Father
6–10
35483 C
36023 G
36517 C
38757 T
Father
--SEA/ αα
6-10-13.fsa
Fetus
6–10
35483 T/C
36023 G
36517 A/C
38757 T
Fetus
αα/ αα

B

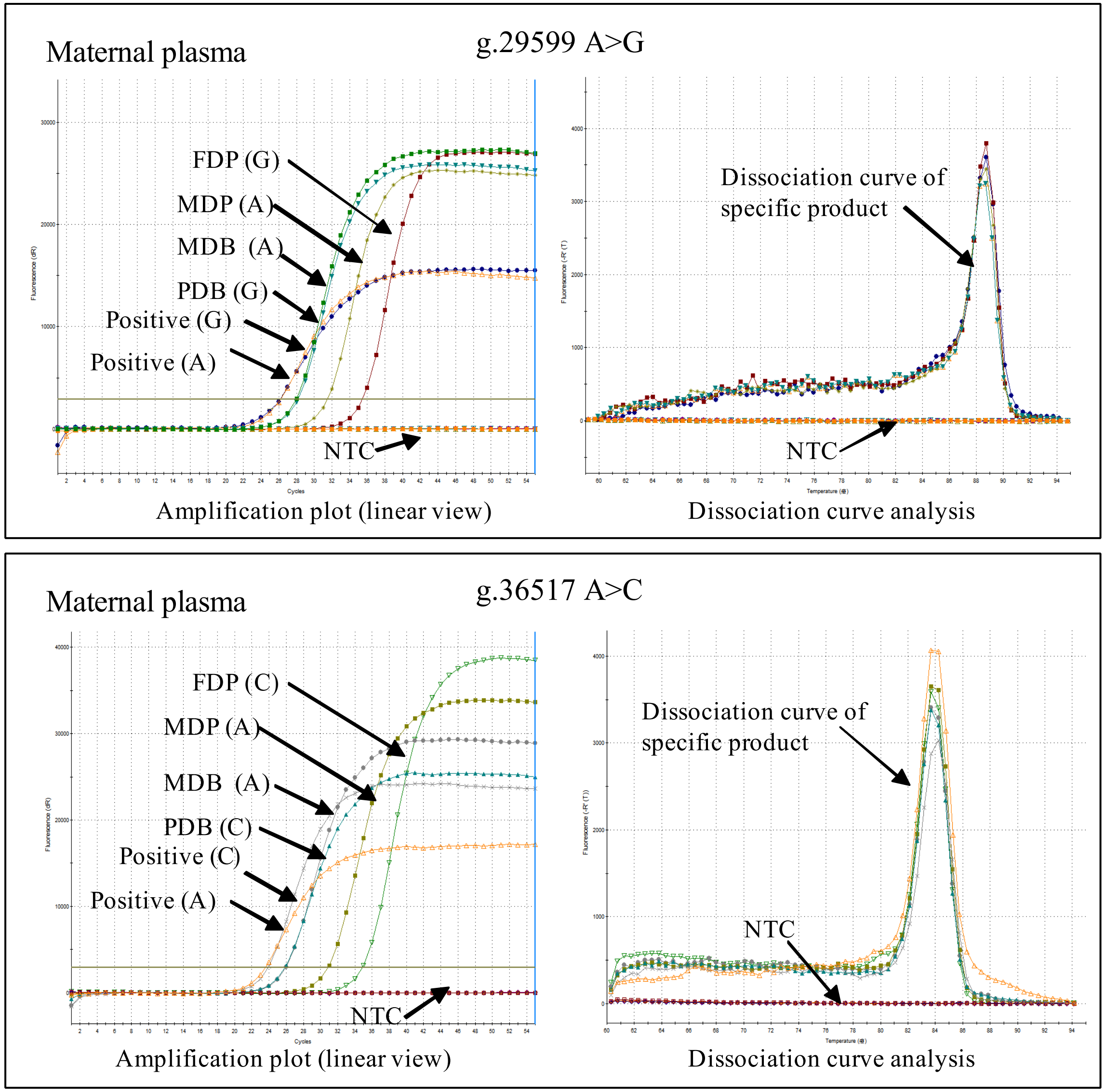

C

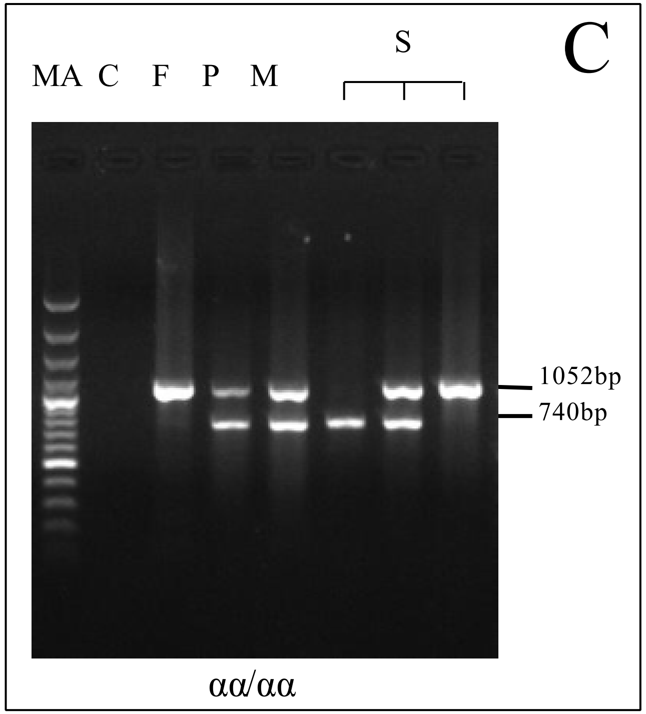

	$C_{T,Maternal}$	$C_{T,Paternal}$	$\triangle C_{T,(P-M)}$
g.29599 A>G	31.52	35.41	3.89
g.36517 A>C	30.96	34.86	3.90

Family 7

A

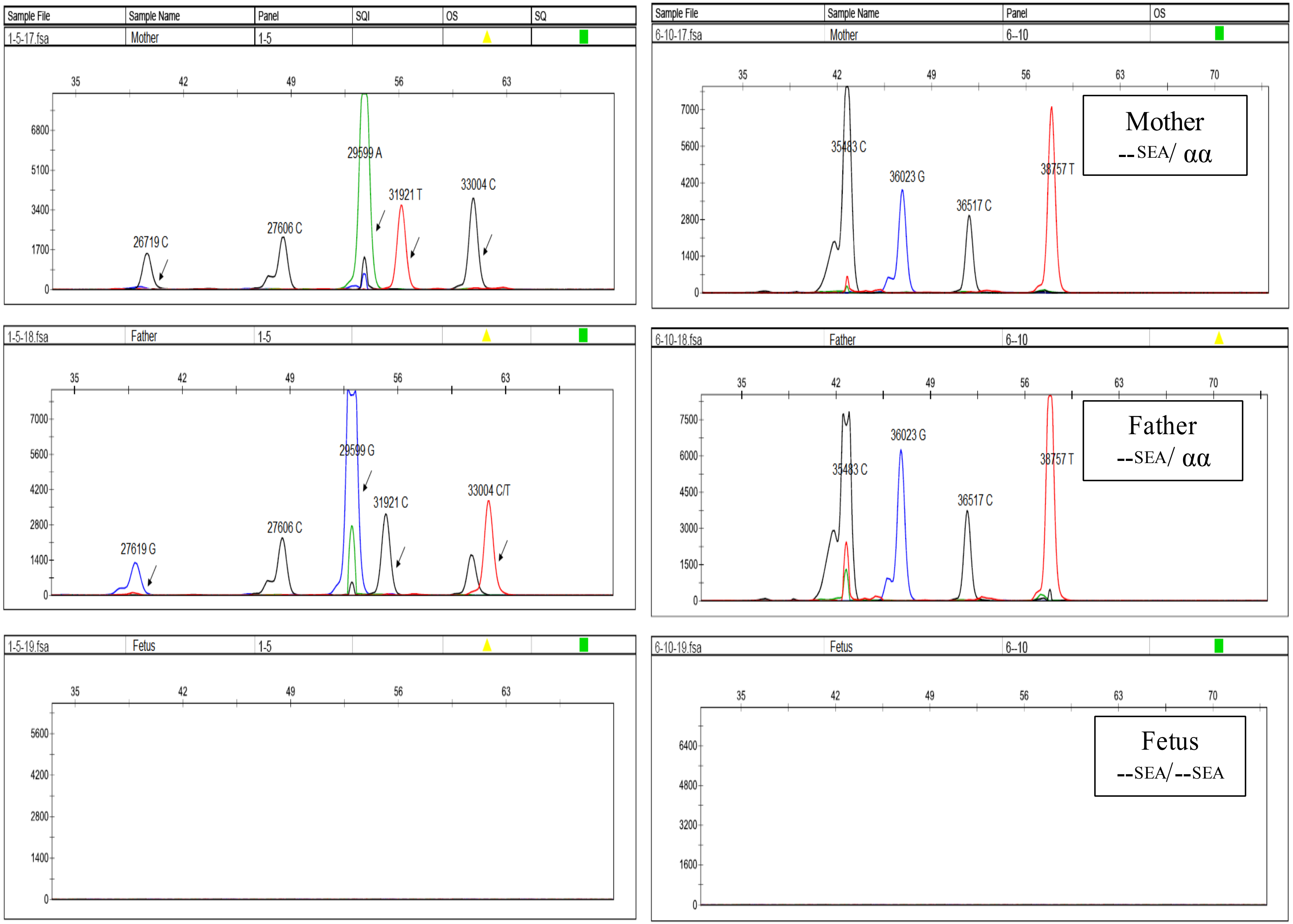

Sample File
Sample Name
Panel
SQI
OS
SQ
1-5-17.fsa
Mother
1-5
26719 C
27606 C
29599 A
31921 T
33004 C
1-5-18.fsa
Father
1-5
27619 G
27606 C
29599 G
31921 C
33004 C/T
1-5-19.fsa
Fetus
1-5
6-10-17.fsa
Mother
6–10
35483 C
36023 G
36517 C
38757 T
Mother
--SEA/ αα
6-10-18.fsa
Father
6–10
35483 C
36023 G
36517 C
38757 T
Father
--SEA/ αα
6-10-19.fsa
Fetus
6–10
Fetus
--SEA/--SEA

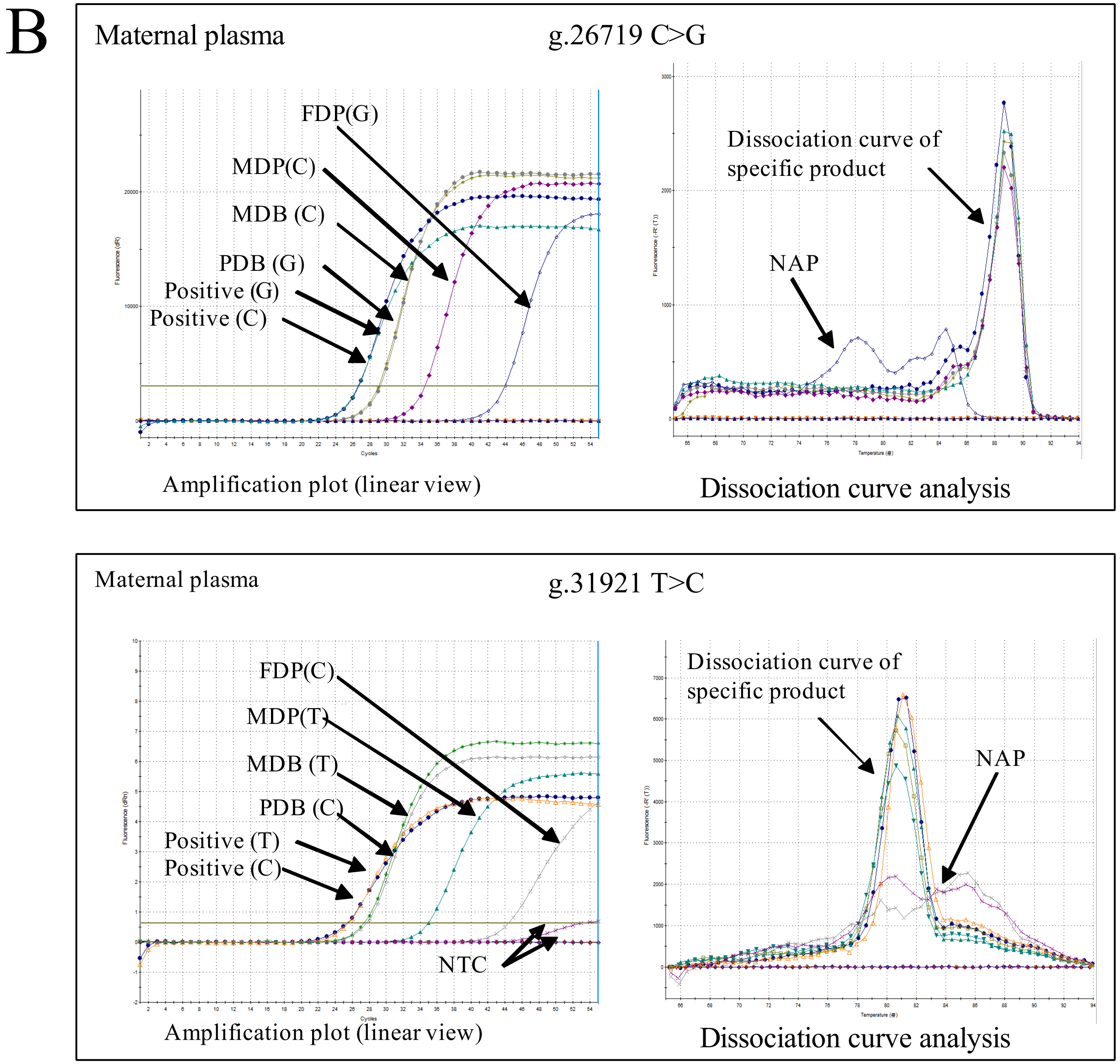

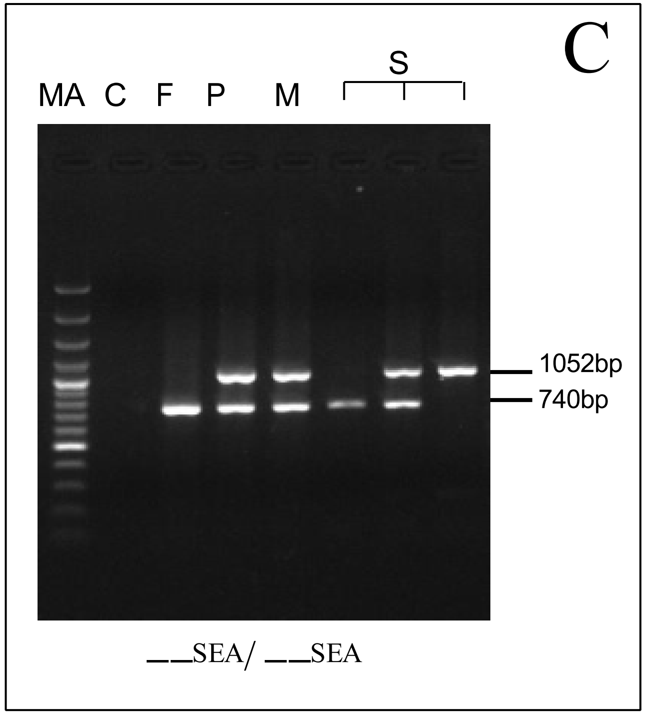

	$C_{T,Maternal}$	$C_{T,Paternal}$	$\triangle C_{T,(P-M)}$
g.26719 C>G	34.53	43.97	9.44
g.31921 T>C	34.94	44.41	9.47

Family 47

Figure S2. Representative NIPD for α-thalassemia in two families performed using our protocol
