# Supplementary material for: Reliable Detection of Paternal SNPs within Deletion Breakpoints for Non-Invasive Prenatal Exclusion of Homozygous α0-Thalassemia in Maternal Plasma
Source: PLoS One. 2011 Sep 29;6(9):e24779. doi: 10.1371/journal.pone.0024779 (PMC3182989; doi:10.1371/journal.pone.0024779)
Supplement: Table S1 — Oligonucleotide sequences for Allele-Specific Real-time PCR. (DOC) [file pone.0024779.s003.doc]

**Table S1. Oligonucleotide sequences for Allele-Specific Real-time PCR.**

| **Marker ID** | **SNP ID** | **Marker position on**  **NG_000006.1** | **Sequence of primers (5’to 3’) ^a^** | **Amplicon (bp)** | **Annealing Temperature (°C)** |
| --- | --- | --- | --- | --- | --- |
| 1 | rs2858935 | g.26719G>C | CO-primer: CCTCCCATGTGCTCCCTCCT | 181 | 62 |
|  |  |  | W-primer: GCCGGGGAACGTAACCAG**A**C |  |  |
|  |  |  | M-primer: GCCGGGGAACGTAACCAG**A**G |  |  |
| 2 | rs75368786 | g.27606C>A | CO-primer: CGAGTTCACCGTGCAAATGC | 141 | 60 |
|  |  |  | W-primer: AGATGGTTCGGGCCTCTGTTTAT**A**G |  |  |
|  |  |  | M-primer: AGATGGTTCGGGCCTCTGTTTAT**A**T |  |  |
| 3 | rs2541675 | g.29599A>G | W-primer: GGTCTGGGAGAAAGTTGG**A**A | 149 | 60 |
|  |  |  | M-primer: GGTCTGGGAGAA AGTTGG**A**G |  |  |
|  |  |  | CO-primer: TGGGTCCAGGGTAAGAATAGT |  |  |
| 4 | rs2974771 | g.31921T>C | CO-primer: GGGAACACAGCTACATCTA | 97 | 58 |
|  |  |  | W-primer: TGAGAATAGGAAGTTGTACA**T**G |  |  |
|  |  |  | M-primer: TGAGAATAGGAAGTTGTACA**T**A |  |  |
| 5 | rs2541669 | g.33004C>T | CO-primer: GCATTTGGAGAGCTGGGGGTGT | 118 | 62 |
|  |  |  | W-primer: CGCGTGGGGTGGTGGG**T**G |  |  |
|  |  |  | M-primer: CGCGTGGGGTGGTGGG**T**A |  |  |
| 6 | rs2238369 | g.35483T>C | W-primer: GTGGAGAGGACCCTGTCA**A**T | 128 | 60 |
|  |  |  | M-primer: GTGGAGAGGACCCTGTCA**A**C |  |  |
|  |  |  | CO-primer: TCTGCTGAAATAACAATGCTCTG |  |  |
| 7 | rs11639532 | g.36023G>A | W-primer: GGGGAAGCATTGCTAAGC**G**G | 160 | 62 |
|  |  |  | M-primer: GGGGAAGCATTGCTAAGC**G**A |  |  |
|  |  |  | CO-primer: GCTTTGAGGAGTGCATCAGGTCA |  |  |
| 8 | rs2858942 | g.36517A>C | CO-primer: CTCCTCCACCTCCTCCACCTAA | 95 | 60 |
|  |  |  | W-primer: GTGGACGAGGCATTCAAG**C**T |  |  |
|  |  |  | M-primer: GTGGACGAGGCATTCAAG**C**G |  |  |
| 9 | rs3760046 | g.38757T>C | CO-primer: CTTTTCAGATTCAATGCAGGTTT | 157 | 60 |
|  |  |  | W-primer: ACTTCTCTGACCTACCCAC**G**A |  |  |
|  |  |  | M-primer: ACTTCTCTGACCTACCCAC**G**G |  |  |

^a^ To prevent amplification, a further mismatch with the target sequence had to be introduced at the second nucleotide from the 3’ end of the primer (bold letter).

Abbreviations: W, wild type allele; M, mutant allele; CO, common.
