# Supplementary material for: Reliable Detection of Paternal SNPs within Deletion Breakpoints for Non-Invasive Prenatal Exclusion of Homozygous α0-Thalassemia in Maternal Plasma
Source: PLoS One. 2011 Sep 29;6(9):e24779. doi: 10.1371/journal.pone.0024779 (PMC3182989; doi:10.1371/journal.pone.0024779)
Supplement: Table S2 — The location of nine SNP markers within (−−SEA) deletion breakpoint regions. (DOC) [file pone.0024779.s004.doc]

**Table S2. The location of nine SNP markers within（−−^SEA^）deletion breakpoint regions.**

| **Marker ID** | **SNP ID** | **Marker position on NG_000006.1** | **Location** |
| --- | --- | --- | --- |
| 1 | rs2858935 | g.26719G>C | in the upstream of *HBM* gene |
| 2 | rs75368786 | g.27606C>A | in the untranslated region of exon 3 of *HBM* gene |
| 3 | rs2541675 | g.29599A>G | within the exon 1 of *HBAP1* gene |
| 4 | rs2974771 | g.31921T>C | between X2 box and Y2 box |
| 5 ^a^ | rs2541669 | g.33004C>T | within the Z2 box |
| 6 ^a^ | rs2238369 | g.35483T>C | within the X1 box |
| 7 | rs11639532 | g.36023G>A | between X1 box and Y1 box |
| 8 | rs2858942 | g.36517A>C | between Y1 box and Z1 box |
| 9 | rs3760046 | g.38757T>C | between *HBA1*gene and *HBQ1*gene |

Abbreviations: *HBM*, hemoglobin mu; *HBAP1*, hemoglobin pseudoalpha 1; *HBA1*, hemoglobin alpha 1; *HBQ1*, hemoglobin theta 1.
