# Supplementary material for: Reliable Detection of Paternal SNPs within Deletion Breakpoints for Non-Invasive Prenatal Exclusion of Homozygous α0-Thalassemia in Maternal Plasma
Source: PLoS One. 2011 Sep 29;6(9):e24779. doi: 10.1371/journal.pone.0024779 (PMC3182989; doi:10.1371/journal.pone.0024779)
Supplement: Table S3 — The quantification results of maternal plasma DNA by Real-time Quantitative-PCR. (DOC) [file pone.0024779.s005.doc]

**Table S3. The quantification results of maternal plasma DNA by Real-time Quantitative-PCR.**

| **Case No.** | **Maternal age (year)** | **Gestational age (week)** | **Total cfDNA concentration (genome equivalents/mL)** | **Fetal DNA concentration (genome equivalents/mL)** |
| --- | --- | --- | --- | --- |
| 1 | 22 | 13.4 | 296.90 | - |
| 2 | 21 | 16.2 | 498.20 | 29.89 |
| 3 | 28 | 23.3 | 1140.75 | 82.13 |
| 4 | 23 | 18.5 | 501.90 | - |
| 5 | 24 | 22.1 | 890.00 | 58.74 |
| 6 | 25 | 20.3 | 785.00 | - |
| 7 | 32 | 19.0 | 748.80 | 35.94 |
| 8 | 24 | 24.1 | 1556.00 | 144.71 |
| 9 | 23 | 22.5 | 860.64 | - |
| 10 | 23 | 21.6 | 641.90 | 42.37 |
| 11 | 33 | 23.1 | 1177.70 | - |
| 12 | 33 | 21.1 | 606.00 | - |
| 13 | 26 | 21.0 | 830.00 | 48.97 |
| 14 | 33 | 17.0 | 473.50 | - |
| 15 | 35 | 25.0 | 1254.71 | 138.02 |
| 16 | 21 | 20.5 | 644.30 | 44.46 |
| 17 | 23 | 24.6 | 981.90 | 82.48 |
| 18 | 29 | 15.0 | 388.20 | 20.57 |
| 19 | 36 | 25.0 | 1085.00 | - |
| 20 | 27 | 15.0 | 355.50 | - |
| 21^a^ | 29 | 23.0 | 49940.00 | - |
| 22 | 39 | 21.3 | 536.40 | 30.57 |
| 23 | 25 | 19.6 | 484.00 | - |
| 24 | 24 | 24.1 | 973.70 | - |
| 25 | 23 | 23.3 | 1140.20 | - |
| 26 | 25 | 24.6 | 987.90 | 70.14 |
| 27 | 31 | 22.3 | 684.50 | 39.70 |
| 28 | 24 | 8.5 | 87.80 | - |
| 29 | 26 | 20.2 | 564.30 | 30.47 |
| 30 | 22 | 13.2 | 298.60 | - |
| 31 | 25 | 16.0 | 391.80 | - |
| 32 | 24 | 23.6 | 803.40 | 49.81 |
| 33 | 41 | 21.3 | 721.60 | 38.97 |
| 34 | 25 | 16.1 | 386.70 | - |
| 35 | 20 | 24.3 | 724.80 | 50.74 |
| 36 | 28 | 16.0 | 421.00 | - |
| 37 | 33 | 24.0 | 943.20 | 69.80 |
| 38^a^ | 32 | 22.0 | 53300.00 | - |
| 39 | 22 | 18.0 | 464.70 | - |
| 40 | 40 | 22.0 | 754.60 | 42.26 |
| 41 | 21 | 22.0 | 798.80 | 49.53 |
| 42 | 31 | 19.0 | 591.00 | - |
| 43 | 30 | 19.0 | 567.40 | 25.53 |
| 44 | 35 | 19.3 | 550.60 | - |
| 45 | 25 | 20.0 | 669.10 | - |
| 46 | 24 | 19.0 | 613.80 | 30.69 |
| 47 | 22 | 17.4 | 414.30 | 20.89 |
| 48 | 25 | 17.5 | 323.70 | - |
| 49 | 26 | 15.2 | 336.00 | - |
| 50 | 28 | 16.0 | 416.80 | 20.84 |
| 51 | 28 | 15.0 | 324.30 | - |
| 52 | 22 | 16.2 | 468.40 | 26.70 |
| 53 | 26 | 16.0 | 419.60 | 21.82 |
| 54 | 30 | 16.0 | 405.20 | - |
| 55 | 30 | 14.0 | 326.80 | - |
| 56 | 28 | 22.3 | 790.75 | - |
| 57 | 25 | 17.0 | 354.90 | - |
| 58 | 29 | 14.0 | 308.60 | - |
| 59 | 27 | 18.0 | 406.40 | 24.38 |
| 60 | 27 | 17.2 | 386.00 | - |
| 61 | 22 | 20.0 | 696.20 | 43.86 |
| 62 | 21 | 18.5 | 594.60 | 28.54 |
| 63 | 33 | 16.1 | 396.00 | - |
| 64 | 28 | 16.5 | 386.00 | - |
| 65 | 31 | 14.4 | 303.00 | - |
| 66 | 23 | 18.6 | 468.00 | 24.34 |
| 67 | 24 | 15.2 | 354.20 | - |
| Mean | 27.16 | 19.12 | 611.63 | 47.35 |
| SD | 4.89 | 3.61 | 284.50 | 30.49 |
| Median | 26.00 | 19.00 | 550.60 | 39.70 |
| Range | 20-41 | 8.5-25 | 87.8-1556 | 19.89-144.71 |

^a^ Due to mishandling the samples, these two cases resulted in hemolysis. They had to be excluded because the amount of cfDNA was substantially higher than would be expected in maternal samples during pregnancy.
