# Supplementary material for: Reliable Detection of Paternal SNPs within Deletion Breakpoints for Non-Invasive Prenatal Exclusion of Homozygous α0-Thalassemia in Maternal Plasma
Source: PLoS One. 2011 Sep 29;6(9):e24779. doi: 10.1371/journal.pone.0024779 (PMC3182989; doi:10.1371/journal.pone.0024779)
Supplement: Table S4 — Allele specific real-time PCR analysis of nine SNP markers using a serial of artificial model samples. (DOC) [file pone.0024779.s006.doc]

**Table S4. Allele specific real-time PCR analysis of nine SNP markers using a serial of artificial model samples.**

| **P:M** | **First amplification** | | | **Second amplification** | | | **Melting curve analysis results** | | | | | | | | |
| --- | --- | --- | --- | --- | --- | --- | --- | --- | --- | --- | --- | --- | --- | --- | --- |
|  | **C_T_**  **_(Maternal)_** | **C_T_**  **_(Paternal)_** | **ΔC_T_**  **_(paternal-maternal)_** | **C_T_**  **_(Maternal)_** | **C_T_**  **_(Paternal)_** | **ΔC_T_**  **_(paternal-maternal)_** | **g.26719**  **G>C** | **g.27606**  **C>A** | **g.29599**  **A>G** | **g.31921**  **T>C** | **g.33004**  **C>T** | **g.35483**  **T>C** | **g.36023**  **G>A** | **g.36517**  **A>C** | **g.38757**  **T>C** |
| 1:1 | 32.14 | 32.2 | 0.06 | 32.28 | 32.25 | -0.03 | √ | √ | √ | √ | √ | √ | √ | √ | √ |
| 1:2 | 32.21 | 33.48 | 1.27 | 32.24 | 33.55 | 1.31 | √ | √ | √ | √ | √ | √ | √ | √ | √ |
| 1:5 | 32.26 | 34.49 | 2.23 | 32.28 | 34.54 | 2.26 | √ | √ | √ | √ | √ | √ | √ | √ | √ |
| 1:10 | 32.34 | 35.56 | 3.22 | 32.38 | 35.62 | 3.24 | √ | √ | √ | √ | √ | √ | √ | √ | √ |
| 1:20 | 32.46 | 36.17 | 3.71 | 32.52 | 36.18 | 3.66 | √ | √ | √ | √ | √ | √ | √ | √ | √ |
| 1:50 | 32.58 | 37.80 | 5.22 | 32.62 | 37.86 | 5.24 | √ | √ | √ | √ | √ | √ | √ | √ | √ |
| 1:100 | 32.62 | 39.13 | 6.51 | 32.67 | 39.24 | 6.57 | - | - | - | - | - | - | - | - | - |
| 1:500 | 32.73 | 40.54 | 7.81 | 32.80 | 40.68 | 7.88 | - | - | - | - | - | - | - | - | - |
| 1:1000 | 32.92 | 42.84 | 9.92 | 32.97 | 42.96 | 9.99 | - | - | - | - | - | - | - | - | - |
| 1:5000 | 33.02 | 44.14 | 11.12 | 33.11 | 44.38 | 11.27 | - | - | - | - | - | - | - | - | - |
| 1:10000 | 33.20 | 46.40 | 13.20 | 33.40 | 47.02 | 13.62 | - | - | - | - | - | - | - | - | - |

√ The paternally inherited allele can be detected in artificial model samples. P, paternal allele; M, maternal allele.
