# Supplementary material for: Reliable Detection of Paternal SNPs within Deletion Breakpoints for Non-Invasive Prenatal Exclusion of Homozygous α0-Thalassemia in Maternal Plasma
Source: PLoS One. 2011 Sep 29;6(9):e24779. doi: 10.1371/journal.pone.0024779 (PMC3182989; doi:10.1371/journal.pone.0024779)
Supplement: Table S5 — Comparison between the analysis of circulating fetal DNA and invasive procedure (CVS or amniocentesis). (DOC) [file pone.0024779.s007.doc]

**Table S5. Comparison between the analysis of circulating fetal DNA and invasive procedure (CVS or amniocentesis).**

| **Family** | **Genotypes** | | **Nine SNP haplotypes ^a^** | | **Detection of paternally inherited alleles in cffDNA** | | **CVS or amniocentesis** |
| --- | --- | --- | --- | --- | --- | --- | --- |
|  | **Mother** | **Father** | **Mother** | **Father** | **Fetal DNA analysis** | **Hb Bart’s excluded** |  |
| 1 | αα/--^SEA^ | αα/--^SEA^ | **C**CATCTGAT | **G**CATCTGAT | Detected | Yes | αα/αα |
| 2 | αα/--^SEA^ | αα/--^SEA^ | **C**CATCTGAT | **G**CATCTGAT | ND ^b^ | No | --^SEA^ /--^SEA^ |
| 3 | αα/--^SEA^ | αα/--^SEA^ | GC**GCTC**G**CT** | GC**ATCT**G**AC** | Detected | Yes | αα/--^SEA^ |
| 4 | αα/--^SEA^ | αα/--^SEA^ | **G**CATCT**A**AT | **C**CATCT**G**AT | ND | No | --^SEA^ /--^SEA^ |
| 5 | αα/--^SEA^ | αα/--^SEA^ | **C**CG**T**TC**A**CT | **G**CG**C**TC**G**CT | Detected | Yes | αα/αα |
| 6 | αα/--^SEA^ | αα/--^SEA^ | **C**C**G**T**TCAC**T | **G**C**A**T**CTGA**T | Detected | Yes | αα/--^SEA^ |
| 7 | αα/--^SEA^ | αα/--^SEA^ | GC**ATCT**G**A**T | GC**GCTC**G**C**T | Detected | Yes | αα/αα |
| 8 | -α^3.7^/--^SEAc^ | αα/--^SEA^ | **G**C**A**T**C---**T | **C**C**G**T**TCAC**T | ND | No | --^SEA^ /--^SEA^ |
| 9 | αα/--^SEA^ | αα/--^SEA^ | CC**A**T**CTGA**T | CC**G**T**TCAC**T | Detected | Yes | αα/--^SEA^ |
| 10 | αα/--^SEA^ | αα/--^SEA^ | **C**C**G**T**TCAC**T | **G**C**A**T**CTGA**T | Detected | Yes | αα/--^SEA^ |
| 11 | αα/--^SEA^ | αα/--^SEA^ | GC**GCTC**G**C**T | GC**ATCT**G**A**T | ND | No | --^SEA^ /--^SEA^ |
| 12 | αα/--^SEA^ | αα/--^SEA^ | GC**G**T**TC**G**CT** | GC**A**T**CT**G**AC** | Detected | Yes | αα/--^SEA^ |
| 13 | αα/--^SEA^ | αα/--^SEA^ | GC**ATCT**G**AC** | GC**GCTC**G**CT** | ND | No | --^SEA^ /--^SEA^ |
| 14 | αα/--^SEA^ | αα/--^SEA^ | **C**CG**T**TC**A**CT | **G**CG**C**TC**G**CT | ND | No | --^SEA^ /--^SEA^ |
| 15 | αα/--^SEA^ | αα/--^SEA^ | GCGCTCGCT | GCGCTCGCT | NI ^d^ | NI ^d^ | αα/αα |
| 16 | αα/--^SEA^ | αα/--^SEA^ | **C**CATCTGAT | **G**CATCTGAT | Detected | Yes | αα/--^SEA^ |
| 17 | αα/--^SEA^ | αα/--^SEA^ | **C**CA**TC**TGA**C** | **G**CA**CT**TGA**T** | Detected | Yes | αα/--^SEA^ |
| 18 | αα/--^SEA^ | αα/--^SEA^ | G**CGTTCAC**T | G**AACCTGA**T | ND | No | --^SEA^ /--^SEA^ |
| 19 | αα/--^SEA^ | αα/--^SEA^ | **G**C**A**T**CTGA**T | **C**C**G**T**TCAC**T | Detected | Yes | αα/--^SEA^ |
| 20 | -α^3.7^/--^SEAc^ | αα/--^SEA^ | GC**AT**T**---**T | GC**GC**T**CGC**T | Detected | Yes | -α^3.7^/αα |
| 21 | αα/--^SEA^ | αα/--^SEA^ | **G**C**G**T**TC**G**C**T | **C**C**A**T**CT**G**A**T | Uncertain ^e^ | Uncertain ^e^ | αα/--^SEA^ |
| 22 | αα/--^SEA^ | αα/--^SEA^ | GC**ATCT**G**A**T | GC**GCTC**G**C**T | Detected | Yes | αα/--^SEA^ |
| 23 | αα/--^SEA^ | αα/--^SEA^ | GC**GCTC**G**C**T | GC**ATCT**G**A**T | Detected | Yes | αα/αα |
| 24 | -α^3.7^/--^SEAc^ | αα/--^SEA^ | G**AAC**C**----**T | G**CGT**C**TGA**T | ND | No | --^SEA^ /--^SEA^ |
| 25 | αα/--^SEA^ | αα/--^SEA^ | CC**A**T**CTGA**T | CC**G**T**TCAC**T | ND | No | --^SEA^ /--^SEA^ |
| 26 | αα/--^SEA^ | αα/--^SEA^ | **C**C**GTCT**G**A**T | **G**C**ACTC**G**C**T | Detected | Yes | αα/αα |
| 27 | αα/--^SEA^ | αα/--^SEA^ | GC**A**T**TCAC**T | GC**G**T**CTGA**T | Detected | Yes | αα/αα |
| 28 | αα/--^SEA^ | αα/--^SEA^ | GC**ATC**TGAT | GC**GCT**TGAT | ND | No | --^SEA^ /--^SEA^ |
| 29 | αα/--^SEA^ | αα/--^SEA^ | **G**C**GCTC**G**C**T | **C**C**ATCT**G**A**T | Detected | Yes | αα/--^SEA^ |
| 30 | αα/--^SEA^ | αα/--^SEA^ | GCGCT**T**G**A**T | GCGCT**C**G**C**T | Detected | Yes | αα/--^SEA^ |
| 31 | αα/--^SEA^ | αα/--^SEA^ | GC**ATCT**G**A**T | GC**GCTC**G**C**T | Detected | Yes | αα/αα |
| 32 | αα/--^SEA^ | αα/--^SEA^ | G**AATCT**G**AC** | G**CGCTC**G**CT** | ND | No | --^SEA^ /--^SEA^ |
| 33 | αα/--^SEA^ | αα/--^SEA^ | GC**ATCT**G**A**T | GC**GCTC**G**C**T | Detected | Yes | αα/--^SEA^ |
| 34 | αα/--^SEA^ | αα/--^SEA^ | GC**GCTC**G**C**T | GC**ATCT**G**A**T | ND | No | αα/--^SEA^ |
| 35 | αα/--^SEA^ | αα/--^SEA^ | GCATCTGAT | GCATCTGAT | NI ^d^ | NI ^d^ | --^SEA^ /--^SEA^ |
| 36 | αα/--^SEA^ | αα/--^SEA^ | **C**C**ATCT**G**A**T | **G**C**GCTC**G**C**T | ND | No | αα/--^SEA^ |
| 37 | αα/--^SEA^ | αα/--^SEA^ | **C**C**ATCT**G**A**T | **G**C**GCTC**G**C**T | Detected | Yes | αα/αα |
| 38 | αα/--^SEA^ | αα/--^SEA^ | G**AA**C**CC**G**C**T | G**CG**C**TT**G**A**T | Uncertain ^e^ | Uncertain ^e^ | αα/--^SEA^ |
| 39 | αα/--^SEA^ | αα/--^SEA^ | GC**ATCT**G**A**T | GC**GCTC**G**C**T | ND | No | αα/--^SEA^ |
| 40 | αα/--^SEA^ | αα/--^SEA^ | **C**C**ATCTGA**T | **G**C**GCTCAC**T | Detected | Yes | αα/--^SEA^ |
| 41 | αα/--^SEA^ | αα/--^SEA^ | GC**GCTC**G**C**T | GC**ATCT**G**A**T | ND | No | αα/--^SEA^ |
| 42 | αα/--^SEA^ | αα/--^SEA^ | GC**GCT**CGCT | GC**ATC**CGCT | ND | No | --^SEA^ /--^SEA^ |
| 43 | αα/--^SEA^ | αα/--^SEA^ | GC**ATCT**G**A**T | GC**GCTC**G**C**T | Detected | Yes | αα/αα |
| 44 | αα/--^SEA^ | αα/--^SEA^ | GCA**T**C**T**G**AC** | GCA**C**C**C**G**CT** | ND | No | --^SEA^ /--^SEA^ |
| 45 | αα/--^SEA^ | αα/--^SEA^ | GC**ATC**CGCT | GC**GCT**CGCT | Detected | Yes | αα/αα |
| 46 | αα/--^SEA^ | αα/--^SEA^ | **G**C**GCTC**G**C**T | **C**C**ATCT**G**A**T | Detected | Yes | αα/--^SEA^ |
| 47 | αα/--^SEA^ | αα/--^SEA^ | **C**C**ATC**CGCT | **G**C**GCT**CGCT | ND | No | --^SEA^ /--^SEA^ |
| 48 | αα/--^SEA^ | αα/--^SEA^ | GCATC**T**G**AC** | GCATC**C**G**CT** | Detected | Yes | αα/αα |
| 49 | αα/--^SEA^ | αα/--^SEA^ | GC**GCTC**G**CT** | GC**ATCT**G**AC** | Detected | Yes | αα/αα |
| 50 | αα/--^SEA^ | αα/--^SEA^ | G**CG**C**TC**G**C**T | G**AA**C**CT**G**A**T | ND | No | --^SEA^ /--^SEA^ |
| 51 | αα/--^SEA^ | αα/--^SEA^ | GC**G**C**TCAC**T | GC**A**C**CTGA**T | ND | No | --^SEA^ /--^SEA^ |
| 52 | αα/--^SEA^ | αα/--^SEA^ | GC**G**T**TC**G**C**T | GC**A**T**CT**G**A**T | ND | No | --^SEA^ /--^SEA^ |
| 53 | αα/--^SEA^ | αα/--^SEA^ | GC**ATCT**G**A**T | GC**GCTC**G**C**T | Detected | Yes | αα/αα |
| 54 | αα/--^SEA^ | αα/--^SEA^ | GC**ATCT**G**A**T | GC**GCTC**G**C**T | Detected | Yes | αα/αα |
| 55 | αα/--^SEA^ | αα/--^SEA^ | **C**CG**T**TCGCT | **G**CG**C**TCGCT | ND | No | αα/--^SEA^ |
| 56 | αα/--^SEA^ | αα/--^SEA^ | GC**A**T**CT**G**AC** | GC**G**T**TC**G**CT** | Detected | Yes | αα/--^SEA^ |
| 57 | αα/--^SEA^ | αα/--^SEA^ | GCGT**TC**G**C**T | GCGT**CT**G**A**T | ND | No | --^SEA^ /--^SEA^ |
| 58 | αα/--^SEA^ | αα/--^SEA^ | **C**CGCTC**A**CT | **G**CGCTC**G**CT | Detected | Yes | αα/--^SEA^ |
| 59 | αα/--^SEA^ | αα/--^SEA^ | **C**CATCTGAT | **G**CATCTGAT | ND | No | --^SEA^ /--^SEA^ |
| 60 | αα/--^SEA^ | αα/--^SEA^ | GC**ATCT**G**A**T | GC**GCTC**G**C**T | ND | No | --^SEA^ /--^SEA^ |
| 61 | αα/--^SEA^ | αα/--^SEA^ | **G**CGCTC**G**CT | **C**CGCTC**A**CT | ND | No | --^SEA^ /--^SEA^ |
| 62 | αα/--^SEA^ | αα/--^SEA^ | GC**A**T**CT**G**A**T | GC**G**T**TC**G**C**T | ND | No | αα/--^SEA^ |
| 63 | αα/--^SEA^ | αα/--^SEA^ | **G**CGCTCGCT | **C**CGCTCGCT | ND | No | αα/--^SEA^ |
| 64 | αα/--^SEA^ | αα/--^SEA^ | GC**GCTT**G**AC** | GC**ATCC**G**CT** | Detected | Yes | αα/--^SEA^ |
| 65 | αα/--^SEA^ | αα/--^THAI f^ | G**A**A**C**CTGAT | G**C**A**T**CTGAT | ND | No | αα/--^SEA^ |
| 66 | αα/--^SEA^ | αα/--^SEA^ | GC**ATCT**G**A**T | GC**GCTC**G**C**T | Detected | Yes | αα/αα |
| 67 | αα/--^SEA^ | αα/--^SEA^ | GC**ATCT**G**A**T | GC**GCTC**G**C**T | ND | No | αα/--^SEA^ |

^a^ Each of the informative nucleotides involved in nine SNPs tested are highlighted with blue bold letter.

^b^ ND: not detected.

^c^ The genotype of these three samples from mothers is−−^SEA^/−α^3.7^.

^d^ Two families are excluded due to lack of informative SNP markers. UI: Uninformative.

^e^ Two cases are classified as uncertain, due to mishandling the samples.

^f^ This at-risk heterozygous couple carries different deletions, mother is a (−−^SEA^) deletion carrier and father is a (−−^THAI^) deletion carrier.
